# *Porphyromonas gingivalis*-induced miR-132 regulates TNFα expression in THP-1 derived macrophages

**DOI:** 10.1186/s40064-016-2363-6

**Published:** 2016-06-17

**Authors:** Mi Hee Park, Eunjoo Park, Hyung-Joon Kim, Hee Sam Na, Jin Chung

**Affiliations:** Department of Oral Microbiology, School of Dentistry, Pusan National University, Yangsan-si, Gyeongsangnam-do 626-870 South Korea; Department of Oral Physiology, School of Dentistry, Pusan National University, Yangsan-si, 626-870 South Korea

**Keywords:** *Porphyromonas gingivalis*, miR-132, Inflammation, TNFα

## Abstract

**Background:**

Periodontitis is a chronic inflammatory disease induced by periodontopathogens such as *Porphyromonas gingivalis* (*P. gingivalis*). MicroRNAs (miRNAs) are small single-stranded noncoding RNAs that regulate gene expression at the level of translation. MiRNAs have been reported to be involved in inflammatory processes. In this study, we examined the effects of *P. gingivalis*-induced inflammatory miRNAs expression on TNFα production in THP-1 derived macrophages.

**Results:**

*Porphyromonas gingivalis* induced the expression of miR-132. *P. gingivalis*-induced miR-132 expression was significantly inhibited by TLR2/4 knock-down and NF-κB inhibitor. Additionally, miR-132 antagomir strongly repressed production of TNFα. The expression of NFE2L2 and NFAT5, the putative target genes of miR-132 involved in regulation of TNFα, decreased in response to *P. gingivalis*. Furthermore, miR-132 antagomir rescued *P. gingivalis*-induced suppression of NFE2L2 and NFAT5.

**Conclusions:**

These results suggest that the induction of miR-132 by *P. gingivalis* can modulate the pathogenesis of periodontitis induced via regulatory expression of TNFα.

## Background

Inflammation is central to effective host defense against invading pathogens. Periodontitis, which is one of the most common human diseases, is an infection-driven chronic inflammatory disease of periodontium and the major cause of tooth loss. Periodontitis is initiated by pathogenic bacteria, such as *Porphyromonas gingivalis* (*P. gingivalis*), *Tannerella forsythia*, and *Treponema denticola* (Byrne et al. 2009; Socransky and Haffajee 1992). *P. gingivalis* is a gram negative anaerobic short rod that expresses various virulence factors, including outer membrane vesicles, lipopolysaccharides (LPS), fimbriae, lipoproteins, and gingipains (Pathirana et al. 2000; Zhou and Amar 2006). *P. gingivalis* modulates the expression of pro-inflammatory cytokines, such as interleukin (IL)-1β, IL-6, IL-8, and TNF-α in human and murine monocytes and macrophages (Agarwal et al. 1995; Park et al. 2014a). The differential regulation of inflammatory cytokines in response to infection is important for recruitment of immune cells in periodontitis tissue and required for orchestration or activation of innate and adaptive immune response.

MicroRNAs (miRNAs) are small single-stranded noncoding RNAs (18–25 nt) that post-transcriptionally regulate gene expression by binding to the 3′ untranslated region (UTR) of target mRNA, leading to mRNA degradation and translational repression (Stoecklin-Wasmer et al. 2012). More than 1000 human miRNAs have been identified in mammals to date (miRBasev.18) (Dai and Ahmed 2011), and they have been found to be associated with diverse biological processes including cell differentiation/proliferation, metabolism, tumorigenesis, and immunity (Ambros 2004; Gregory and Shiekhattar 2005; Taganov et al. 2007).

Recently, several miRNAs related to periodontal inflammation have been reported. In a previous study, we reported differential miRNA expression in healthy and periodontitis tissues (Lee et al. 2011). MiR-132 and miR-146 are inflammatory miRNAs related to bacterial infection. MiR-146 induces a negative feedback mechanism in response to bacterial products-induced TLR signaling to prevent excessive inflammation via suppression of IL-1β, IL-6, IL-8, and TNF-α (Marques-Rocha et al. 2015; Staedel and Darfeuille 2013). Overexpression of miR-132 is sufficient to promote activation of NF-κB and production of IL-8 and MCP-1 (Strum et al. 2009). Although these studies shed some light on the relationship between inflammation and miRNA, bacterial infection-induced miR132 function on inflammatory response is poorly understood. Furthermore, limited data describing the role of miRNAs related to periodontitis or periodontopathogens and the effects of *P. gingivalis* on the expression of various types of miRNA are available.

In the present study, we investigated whether *P. gingivalis* can affect the expression of inflammatory miRNAs involved in networks that control innate immunity and extend beyond TLR regulation in THP-1 derived macrophages. We found that *P. gingivalis* induced miR-132 via TLR2/4 and NF-κB signaling. Moreover, the inhibition of miR-132 expression strongly suppressed the production of TNF-α. The expression of NFE2L2 and NFAT5, potential target molecules of miR-132, decreased in response to *P. gingivalis* and was recovered by miR-132 antagomir treatment. These results suggest the role of miR-132 in the pathogenesis of periodontitis induced by *P. gingivalis.*

## Methods

### Bacterial culture

*Porphyromonas gingivalis* (strain 381) wild type and various mutants (MT10, MT10W, DPG) were grown in GAM broth (Nissui Pharmaceutical, Japan) with 5 mg/ml hemin and 0.5 mg/ml vitamin K under anaerobic conditions at 37 °C. An OD of 1.0 (650 nm) was determined to correlate to 10^9^ CFU/ml. The bacteria were washed and suspended in serum free-RPMI media to infect THP-1 derived macrophages at various multiplicity of infections (MOIs).

### Cell lines

THP-1 cells were cultured in RPMI 1640 medium with 10 % heat-inactivated fetal bovine serum (FBS; Gibco), 100 U of penicillin/ml, and 100 μg of streptomycin/ml at 37 °C in a 5 % CO_2_/95 % air incubator. THP-1 cells were differentiated into macrophage-like cells with 50 ng/ml of Phorbol 12-mystristate 13-acetate (PMA; Sigma).

### Real-time quantitative reverse-transcriptase polymerase chain reaction (qRT-PCR)

Total RNA was extracted with TRIzol (Invitrogen, USA) according to the manufacturer’s instructions. For miRNA analysis, 10 ng of RNA from each sample was used for quantitative stem-loop reverse transcription and real-time PCR (qRT-PCR). Quantification of expression of mature miRNAs was performed using a TaqMan micro-RNA RT kit, TaqMan Universal PCR Master Mix, and gene-specific primers and fluorogenic Taqman probes (Applied Biosystems, USA). MiRNA expression values were calculated using human RNU44 as an endogenous reference. For miRNA target gene analysis, the reverse transcription of total RNA to cDNA was performed using AccuPower RT PreMix (Bioneer Co, South Korea). The mRNA expression levels were quantified by real-time PCR using a Light Cycler instrument (Roche Applied Science, Mannheim, Germany) with SYBR Green PCR Master Mix (Qiagen, USA) according to the manufacturer’s instructions.

### Transient transfection

For RNA interference assay, human siRNAs for TLR2, TLR4, and non-targeting control oligonucleotides were obtained from Qiagen. The siRNA oligonucleotides in this pool were as follows: TLR2, TLR4, and non-targeting control siRNA oligonucleotides. For siRNA experiments in THP-1 cells, cells were seeded in 6-well plates at a density of 1 × 10^6^ cells per well in the presence of 50 nM PMA. The differentiated cells were then transfected with siRNA oligonucleotides (1200 ng) for 48 h using Attractene Transfection Reagent (Qiagen, USA) in 1 ml of RPMI. MiR-132 functional analyses were performed by transfecting synthetic antagomir (20 pmol) in THP-1 cells using Attractene Transfection Reagent.

### NF-κB DNA binding assay

Binding of p65 to the double-stranded NF-κB oligonucleotide was measured using a NF-κB Combo Transcription Factor Assay kit (Cayman Chemical Company, USA).

### Measurement of TNF-α and IL-1β

To determine the amount of TNF-α and IL-1β released into the culture media after *P. gingivalis* stimulation, we analyzed the amount in accordance with the manufacturer’s instructions using an ELISA kit (eBioscience, San Diego, CA, USA).

### Immunoblot analysis

Cells were washed in ice-cold PBS and lysed, after which a total of 50 μg proteins were separated by 15 % SDS-PAGE, transferred to a PDVF membrane and probed with the indicated antibodies. Reactivity was determined using HRP-conjugated anti-mouse, anti-rabbit or anti-mouse IgG secondary antibody (Jackson Immuno Research Laboratories, USA) (diluted 1:5000) and the signals were visualized using Super Signal West Femto maximum sensitivity substrate (Pierce, USA) with a LAS-4000 FUJIFILM Luminescent Image Analyzer (Fujifilm, Japan).

### ASC pyroptosome quantitation in live cells

THP-1/ASC-GFP cells were seeded in 8 well chambers and then primed with PMA (50 nM). THP-1/ASC-GFP cells were transfected with miR-132 antagomir and further challenged with *P. gingivalis*. ASC pyroptosome formation was observed by confocal Laser-Scanning Microscopy (Carl Zeiss, USA) at various time points. At least 400 cells were counted to enumerate the number of cells containing the ASC-GFP pyroptosome at the end of each period. The percentage of cells with ASC pyroptosome was calculated by dividing the number of cells with ASC pyroptosome by the total cells counted.

### Statistics

Statistically significant differences between samples were analyzed with an unpaired, one-tailed Student’s *t* test. The data are shown as the mean ± standard deviation (SD).

## Results

### *Porphyromonas gingivalis* infection up-regulates miR-132 expression in THP-1 derived macrophages

**Fig. 1:**
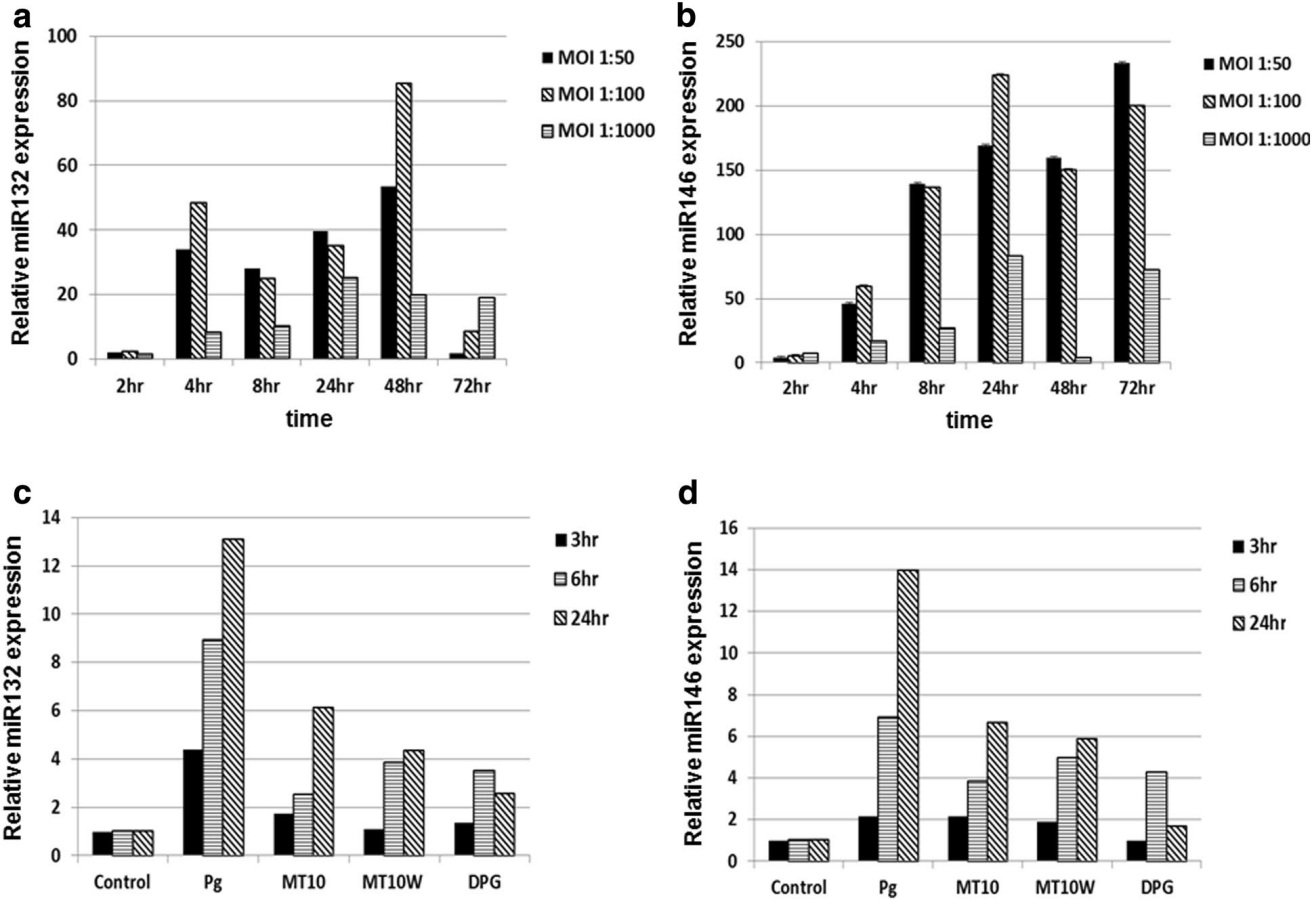
MiR-132 and miR-146 were induced by live *P. gingivalis* infection. THP-1-derived macrophages were infected with live *P. gingivalis* (MOI 50, 100, 1000) for the indicated times. Total RNA was purified from the respective cell pellets and analyzed by qRT-PCR for the expression of miR-132 (**a**) and miR-146 (**b**). THP-1 derived macrophages were infected with various strains of *P. gingivalis* (MOI 100) for the indicated times (**c**, **d**). *MOI* multiplicity of infection

To investigate the relationship between periodontitis and miRNA, we examined the effects of *P. gingivalis* on the expression of inflammatory miRNAs, miR-132 and miR-146 in THP-1 derived macrophages. MiR-132 and miR-146 expression were induced by live *P. gingivalis* infection in a MOI-dependent and time-dependent manner (Fig. 1a, b). To gain more insight into the mechanism by which *P. gingivalis* induces miRNAs expression, the mutant strains tested included MT10 (RgpA-), MT10W (RpgA-, Kgp-) and DPG (fimbriae-). The mutant strains showed less of an increase of expression of miR-132 and miR-146 than wild-type strain (Fig. 1c, d). These results suggest that the virulence factors, gingipain and fimbriae, may be involved in induction of miR-132 to regulate expression of TNFα. We focused on miR-132 for further investigation because the relationship between miR-132 and *P. gingivalis* has not been studied, even though miR-132 is associated with regulation of inflammation.

### *Porphyromonas gingivalis* induces miR-132 expression through TLR2/4 signaling and NF-κB signaling in THP-1 derived macrophages

**Fig. 2:**
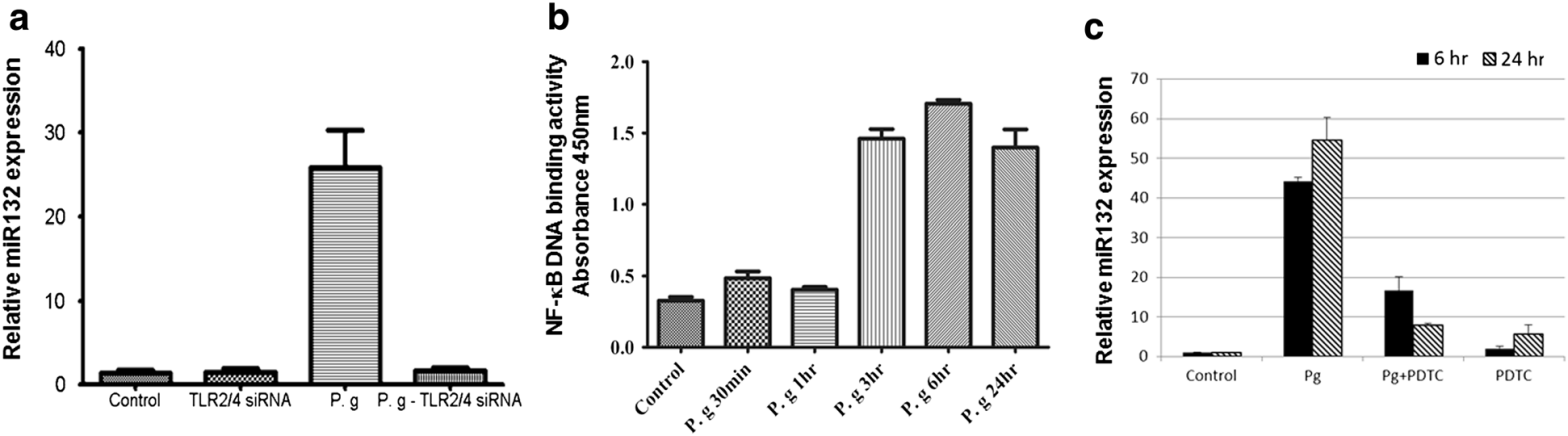
TLR2/4 and NF-κB is required for *P. gingivalis*-induced miR-132 expression. **a** THP-1 cells were transfected with 100 nM siTLR2 and siTLR4 for 48 h, then infected with *P. gingivalis* (MOI 100) for 6 h. **b** THP-1 derived macrophages were infected with *P. gingivalis* (MOI 100) for the indicated times. Nuclear extracts were analyzed for p65 and p50 binding to the NF-κB double-stranded oligonucleotide using the NF-κB Transcription Factor ELISA Assay kit. **c** THP-1 derived macrophages were pretreated with 300 μM PDTC for 1 h, then infected with live *P. gingivalis* (MOI 100) for indicated times, after which the total RNA was purified from the respective cell pellets and analyzed by qRT-PCR for the expression of miR-132. The data represent mean values ± SD (n ≧ 3). *Bar graphs* show the fold increase of protein expression compared with control cells; *columns* show mean values of n ≧ 3 samples; *error bars* show standard deviation

Because *P. gingivalis* activates both TLR2 and TLR4, we investigated the involvement of TLR2 and TLR4 in *P. gingivalis*-induced miRNA expression by RNA interference assay. Knockdown of TLR2 and TLR4 significantly decreased *P. gingivalis*-induced miR-132 expression relative to siRNA control (Fig. 2a). TLR2 and/or TLR4 activate NF-κB for the induction of inflammation. Thus, we examined the involvement of NF-κB in *P. gingivalis*-induced miR-132 expression. The activation of NF-κB, as measured by p65-50 binding to an NF-κB consensus oligonucleotide, was observed at 3 h post-infection (Fig. 2b). The induction of miR-132 expression by *P. gingivalis* was inhibited by pretreatment of the cells with PDTC, an NF-κB inhibitor (Fig. 2c). These results suggest that *P. gingivalis* induces miR-132 through the activation of TLR2/4 and NF-κB.

### The inhibition of miR-132 expression down-regulates the expression of TNFα, but not IL-1β, induced by *P. gingivalis* infection in THP-1 derived macrophages

**Fig. 3:**
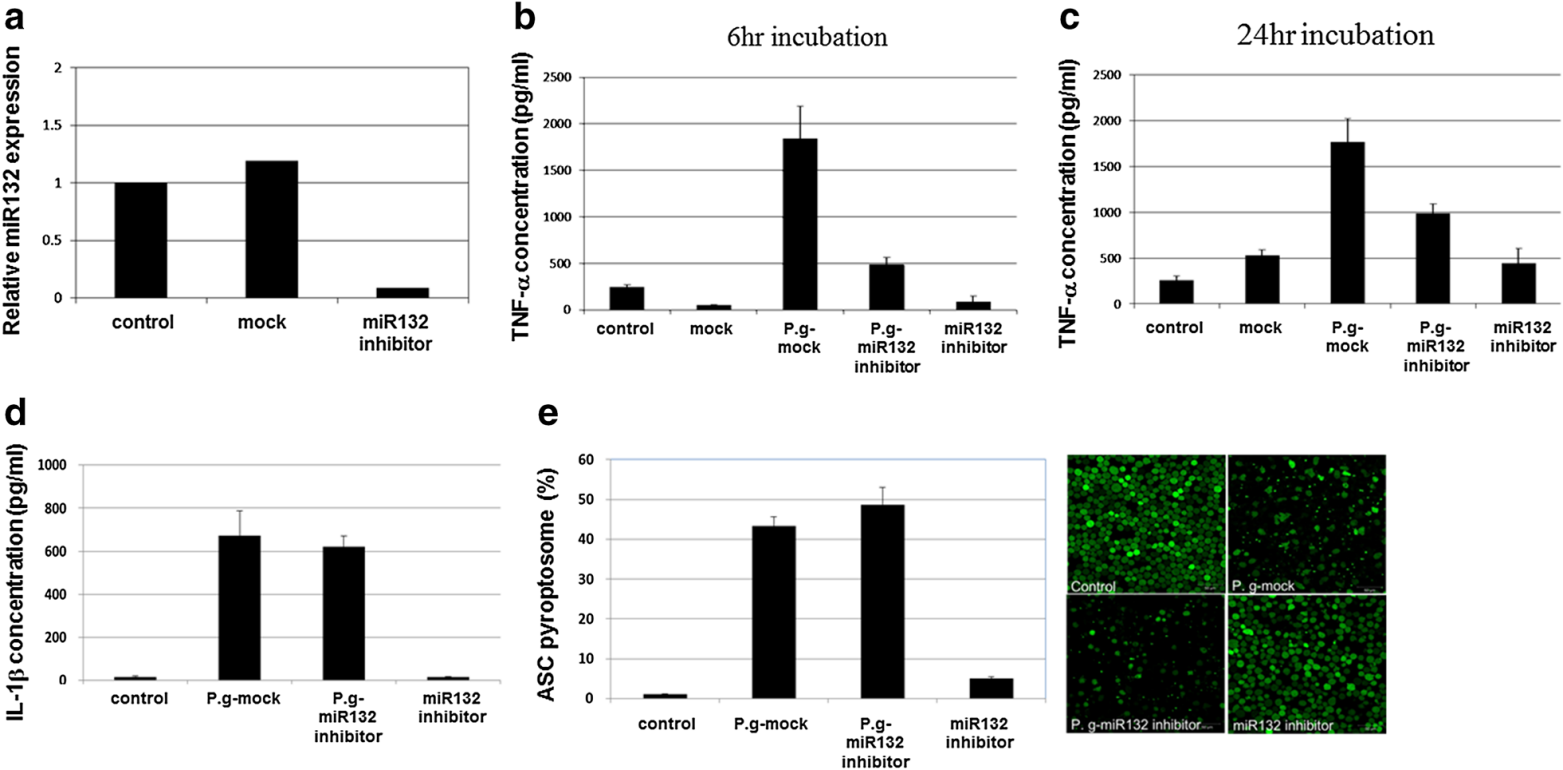
MiR-132 is involved in TNF-α, but not IL-1β, induced by *P. gingivalis* infection in THP-1 derived macrophages. THP cells were transfected with control or miR-132 antagomir (20 pmol). At 24 h post-transfection, RNA was extracted and miR-132 expression was verified by qRT-PCR (**a**). At 24 h post-transfection, THP-1 cells were infected with live *P. gingivalis* (MOI 100) for 6 h (**b**) or 24 h (**c**, **d**). Cell culture supernatants were assayed for human TNF-α (**b**, **c**) and IL-1β (**d**) using ELISA. **e** PMA-primed ASC-GFP stable expressing-THP-1 cells were transfected with control or miR-132 antagomir (20 pmol). At 24 h post-transfection, samples were infected with *P. gingivalis* for 24 h and ASC-pyroptosomes were then observed and photographed by fluorescence confocal microscopy (*right panel*). The *graph on the left* shows the percentages of cells containing the ASC pyroptosome. The percentages of cells containing the ASC pyroptosome were calculated as described in the “Methods” section. *TNFα* tumor necrosis factor. The data represent mean values ± SD (n ≧ 3). *Bar graphs* show the fold increase of protein expression compared with control cells; *columns* show mean values of n ≧ 3 samples; *error bars* show standard deviation

To test the effects of miR-132 on pro-inflammatory cytokine production by live *P. gingivalis* infection, THP-1 cells were transfected with miR-132 antagomir. As shown in Fig. 3a, miR-132 expression in miR-132 antagomir transfected cells showed a 90 % reduction compared with mock transfected cells. MiR-132 antagomir treatment (transfection) resulted in a decrease in *P. gingivalis*–induced TNFα (Fig. 3b, c), but not IL-1β secretion (Fig. 3d). Because the production of mature IL-1β required the activation of inflammasome, we investigated the effects of miR-132 on inflammasome activation. As shown in Fig. 3e, miR-132 antagomir transfection did not affect the formation of ASC pyroptosome in response to *P. gingivalis* infection. These results indicate that *P. gingivali*s induces miR-132 expression and subsequently enhances the expression of TNFα.

### Inhibition of miR-132 expression restored the expression of NFE2L2 and NFAT5 decreased by *P. gingivalis* infection in THP-1 derived macrophages

**Fig. 4:**
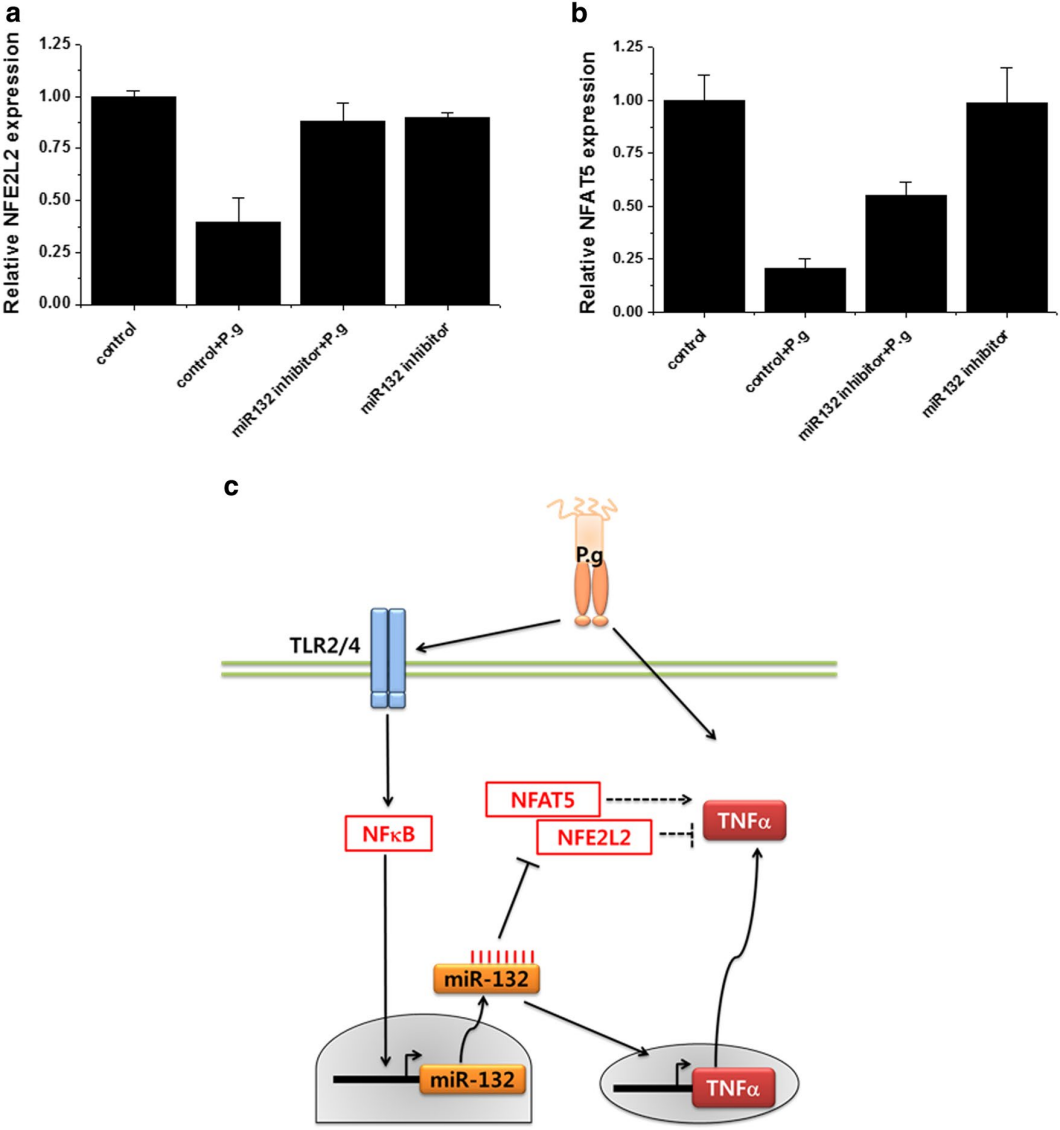
MiR-132 regulates the expression of NFE2L2 and NFAT5 induced by *P. gingivalis* infection in THP-1 cells. **a**, **b** THP cells were transfected with control or miR-132 antagomir (50 pmol). At 24 h post-transfection, THP-1 cells were differentiated by PMA for 24 h, RNA was extracted and the expression of NFE2L2 and NFAT5 was verified by qRT-PCR. The data represent mean values ± SD (n ≧ 3). *Bar graphs* show the fold increase of protein expression compared with control cells; *columns* show mean values of n ≧ 3 samples; *error bars* show standard deviation. **c** A proposed model of *P. gingivalis*-induced miR-132 regulates TNFα in THP-1-derived macrophages

To identify potential targets of miR-132 involved in inflammatory response, we used the online bioinformatics program TargetScan (http://www.targetscan.org). NFE2L2 and NFAT5 were identified as predicted targets of miR-132. To test whether miR132 would modulate NFE2L2 and NFAT5 as well as inflammatory cytokines in *P. gingivalis*-infected THP-1 derived macrophages, THP-1 cells were transfected with miR-132 antagomir. As shown Fig. 4a and b, *P. gingivalis* decreased NFE2L2 and NFAT5 expression and miR-132 antagomir up-regulated NFE2L2 and NFAT5 expression in *P. gingivalis*-infected THP-1 derived macrophages. These data suggest the possible direct repression of NFE2L2 and NFAT5 by miR-132.

## Discussion

In this study, we investigated the effects of *P. gingivalis* on the expression of inflammatory miRNAs, miR-132 and miR-146, and the role of miR-132 in live *P. gingivalis*-challenged THP-1-derived macrophages. *P. gingivalis* induced miR-132 expression via TLR2/4 and NF-κB signaling, and the enhanced miR-132 expression was closely associated with the increased production of TNFα (Fig. 4c).

MiRNAs are abundant in the human genome and control various important cellular processes, including proliferation, differentiation, metabolism, apoptosis and inflammation (Ambros 2004; Zhang and Su 2009). A single miRNA is loaded into the RNA-induced silencing complex (RISC) and guides this complex to the 3′ UTRs of target mRNA, leading to suppressed target protein expression. Several lines of evidence suggest that miRNAs play critical roles in a number of chronic inflammatory diseases (Plank et al. 2013; Zhang et al. 2014). In innate immune response, specific miRNAs can be up- or down-regulated by inflammatory stimuli. The miRNAs significantly linked to inflammatory response were miR-146, miR-155, miR-21, miR-9 and miR-132 (Dai and Ahmed 2011; Schetter et al. 2010), among which miR-132, miR-146, and miR-155 are inflammatory miRNAs. miR-146 and miR-155, but not miR132, are known to be associated with periodontal disease (Xie et al. 2011). MiR-146 expression increased in periodontal-disease gingival tissue (Lee et al. 2011; Xie et al. 2011) and *P. gingivalis*-LPS induced miR-146 expression in THP-1 cells/THP-1-derived macrophages (Honda et al. 2012) and human gingival fibroblasts (Xie et al. 2013). Honda et al. (2012) suggested that *P. gingivalis*-LPS-induced miR-146 expression did not modulate proinflammatory cytokines production. Consistent with the above reports, we found that *P. gingivalis* induced miR-146 in THP-1-derived macrophages. Although these studies demonstrated the involvement of miRNA in periodontitis, the association of miR-132 with periodontitis has not yet been reported.

MiR-132 is well characterized in brain neurons and plays an important role in inflammation, angiogenesis, and neural development (Magill et al. 2010; Mulik et al. 2012; Shaked et al. 2009). MiR-132 modulates neuronal survival in AD neurodegeneration (Wong et al. 2013) and attenuates inflammation in the mouse brain by targeting acetylcholinesterase (Shaked et al. 2009). In innate immune response, miR-132 plays important roles in TLR2 ligand–induced tolerance and cross-tolerance by targeting IRAK4 (Nahid et al. 2013). Nevertheless, the function of miR-132 has been described in only a few studies and is still poorly understood in innate immune response. Our data are the first to reveal the relationship between miR-132 and *P. gingivalis*-mediated inflammatory response in THP-1-derived macrophages. The bacterial cell surface components facilitate growth, nutrient acquisition, colonization, biofilm formation and evasion of host defense (Yoshimura et al. 2009). Several reports suggest that gingipains is critical for proinflammatory response in periodontal disease progression (Kataoka et al. 2014; Tancharoen et al. 2015). Fimbriae are potent stimulators of inflammation (Takahashi et al. 2006), and *P. gingivalis* fimbriae significantly induced TNFα production via TLR2 in macrophages (Aoki et al. 2010). Interestingly, our data showed that gingipain mutants (MT10 and MT10 W) and fimbriae mutant (DPG) of *P. gingivalis* resulted in less increase of miR-132 compared to wild-type. Even though involvement of gingipain and fimbriae in miR132 expression has not yet been investigated, these reports coupled with our results support the possibility that gingipain and fimbriae contribute to production of TNFα via miR-132 induction.

In our study, live *P. gingivalis* induced miR-132, and its regulation was dependent on TLRs. TLRs recognize pathogens and activate signaling pathways including immune and inflammatory response to regulate cytokines, chemokines, and adhesion molecules and eliminate pathogens. Several reports recently revealed that TLR signaling modulates various miRNAs (Nahid et al. 2011; O’Neill et al. 2011). miR-146, miR-155 and miR-21 have been central in many miRNA studies due to their expression levels following TLR activation (Quinn and O’Neill 2011). Furthermore, miR-9, miR-147, miR-27b, miR-223, and let-7e are induced by TLR stimulus or pathogen infection (He et al. 2014). However, only a few studies have investigated TLR-mediated miR-146 expression in periodontal disease (Honda et al. 2012; Xie et al. 2013). Indeed, this study is the first study to suggest *P. gingivalis* induces miR-132 via TLR signaling.

The expression of most TLR-responsive miRNAs described to date depends on NF-κB activity. MiR-155 positively regulates inflammation through the TLR-NF-κB pathway in response to *Francisella* (Cremer et al. 2009). MiR-9, miR-146, miR-181b and miR-21 are also directly regulated through the NF-κB-dependent pathway (Ma et al. 2011). Conversely, miR-29b, let-7i, miR-98, miR-107, miR-27a, and miR-532-5p are negatively regulated by the TLR-NF-κB pathway (He et al. 2014). We found that inhibition of TLR signaling or NF-κB abolished *P. gingivalis*-induced miR-132 expression. Although this suggests that miR-132 may be directly regulated by the TLR2/4-NF-κB signaling pathway, further studies are needed to better understand the exact mechanism.

Cytokines are key modulators of inflammatory response classified as proinflammatory (IL-1, IL-6, IL15 and TNFα) or anti-inflammatory (IL-4, IL-10, TFGβ and IFNα) (Schetter et al. 2010). Among various cytokines, TNFα affects many functions such as inflammation, tumor promotion, and anti-microbial immunity (Sedger and McDermott 2014). The expression of TNFα was higher in periodontitis patients than healthy controls, and periodontal bacteria stimulate expression of TNFα (Preshaw and Taylor 2011). Consistent with the above data, we previously reported that TNFα was higher in the GCF of periodontitis patients and *P. gingivalis*-LPS induced gene expression and protein synthesis of TNFα (Han et al. 2005; Park et al. 2014a). Similar to TNFα, IL-1β is a pro-inflammatory cytokine critical to inflammatory response against pathogens that is well known for involvement in periodontal disease. We recently found that the levels of IL-1β and inflammasome components increased in periodontitis patients and *P. gingivalis* induced IL-1β release via TLR2/TLR4-NLRP3/AIM2 inflammasome-caspase-1 pathway activation (Park et al. 2014b). In the current study, we found that miR-132 positively regulates TNFα, but not IL-1β. Furthermore, miR-132 did not affect inflammasome activation. However, some studies suggest that IL-1β is a target of miR-132, in contrast to our results (Fen et al. 2014; Hanieh and Alzahrani 2013). The precise reasons for this difference are not clear, but these results may be dependent on cell type and stimuli; accordingly, these mechanisms should be further investigated.

Nuclear factor E2-related factor 2 (NFE2L2 or Nrf2) is a master transcription factor essential for protection from oxidative stress via modulation of ARE-dependent genes, including hemo oxygenase-1 (HO-1), a well-known anti-inflammatory enzyme (Hu et al. 2010; Kim et al. 2010). Park et al. recently reported that the anti-inflammatory effect of Schisandrin on *P. gingivalis*-LPS-stimulated macrophages was due to induction of HO-1 and activation of Nrf-2. Inhibition of miR-132 restored the ochartoxin-driven decrease of Nrf2 and HO-1 expression as well as the increase in ROS production (Stachurska et al. 2013). Our study also found that inhibition of miR-132 rescued *P. gingivalis*-mediated deduction of Nrf-2 expression. Moreover, our results indicated that *P. gingivalis*-induced production of TNFα may be enhanced by miR-132 via miR-132-mediated suppression of Nrf2 expression and anti-inflammatory effect of Nrf2. Nuclear factor of activated T cells 5 (NFAT5) is a transcription factor involved in immune response by regulating TNFα expression via direct binding to TNF promoter and enhancing TNF transcription (Esensten et al. 2005). Although NFAT5 positively regulates TNFα, our results showed that *P. gingivalis* and *P. gingivalis*-induced miR-132 negatively regulated NFAT5 expression. We assume that these results occurred due to a negative-feedback loop in which *P. gingivalis*-induced miR-132 attenuated pro-inflammatory effects of *P. gingivalis* by targeting NFAT5 expression. Since we do not yet fully understand how miR-132-targeted NFAT5 affects the expression of TNFα by *P. gingivalis*, the exact involvement of miR-132 and NFAT5 in *P. gingivalis*-induced inflammation will be confirmed later.

## Conclusions

In this study, live *P. gingivalis* infection induced miR-132 via TLR signaling and activation of NF-κB. Furthermore, inhibition of miR-132 expression strongly repressed the production of TNFα and increased NFE2L2 and NFAT5. This is the first study to describe the novel role of miR-132 in the pathogenesis of *P. gingivalis*. These results support the concept that miR-132 modulates TNFα via inhibition of its target genes, which may provide a new window of opportunity to investigate therapeutic intervention for *P. gingivalis*-induced TNFα associated diseases such as periodontitis.
